# Multilevel evolution shapes the function of NB-LRR encoding genes in plant innate immunity

**DOI:** 10.3389/fpls.2022.1007288

**Published:** 2022-10-27

**Authors:** Maria Raffaella Ercolano, Daniela D’Esposito, Giuseppe Andolfo, Luigi Frusciante

**Affiliations:** Department of Agricultural Sciences, University of Naples ‘Federico II’, Portici, Italy

**Keywords:** NBS-LRR genes, functional domain, genome organization, regulatory elements, plant receptor genes network, innate immunity

## Abstract

A sophisticated innate immune system based on diverse pathogen receptor genes (PRGs) evolved in the history of plant life. To reconstruct the direction and magnitude of evolutionary trajectories of a given gene family, it is critical to detect the ancestral signatures. The rearrangement of functional domains made up the diversification found in PRG repertoires. Structural rearrangement of ancient domains mediated the NB-LRR evolutionary path from an initial set of modular proteins. Events such as domain acquisition, sequence modification and temporary or stable associations are prominent among rapidly evolving innate immune receptors. Over time PRGs are continuously shaped by different forces to find their optimal arrangement along the genome. The immune system is controlled by a robust regulatory system that works at different scales. It is important to understand how the PRG interaction network can be adjusted to meet specific needs. The high plasticity of the innate immune system is based on a sophisticated functional architecture and multi-level control. Due to the complexity of interacting with diverse pathogens, multiple defense lines have been organized into interconnected groups. Genomic architecture, gene expression regulation and functional arrangement of PRGs allow the deployment of an appropriate innate immunity response.

## Overview

A large assortment of innate immunity receptors, able to perceive pathogen invasion and to activate a defense response, have evolved in plants.

Cell surface receptors, such as receptor-like proteins (RLPs) and receptor-like kinases (RLKs), are mainly involved in monitoring the extracellular space to detect exogenous (microbe-associated molecular patterns, MAMPs) or endogenous elicitors (damage-associated molecular patterns, DAMPs) generated by plant pathogens (Heil and Land, 2014; Tanaka and Heil, 2021). A typical RLP structure is composed of an extracellular domain, responsible in MAMP/DAMP perception, a single-pass transmembrane region and a short cytoplasmic tail (Thomas et al., 1997). RLK are structurally similar to RLPs except for the presence of an intracellular kinase domain instead of the short cytoplasmic tail (Fritz-Laylin et al., 2005). Thanks to the kinase domain, RLKs are able to trigger signaling on their own (Liebrand et al., 2013 and 2014; Gust and Felix, 2014), whilst RLPs need to interact with a protein containing such domain to activate the downstream signaling (Jamieson et al., 2018).

Intracellularly, nucleotide-binding leucine-rich repeat proteins (NB-LRRs or NLRs) can directly or indirectly recognize “effectors,” molecules secreted or delivered by pathogens into the cytoplasm to promote virulence (Van der Hoorn and Kamoun, 2008). NLRs have a stereotypical domain structure that allows them to recognize effectors and activate immunity. The nucleotide-binding (NB) domain, containing an ADP–ATP switch system that regulates the protein ON/OFF state, is the central module of NLR proteins (Takken et al., 2006). In addition, several leucine-rich repeats, that promote pathogen recognition and interact with the NB domain to prevent autoactivation, are found at the C-terminus (Wang et al., 2019). The N-terminal domain, thanks to the Toll/interleukin-1 receptor (TIR), coiled-coil (CC), resistance to powdery mildew 8 (RPW8) or similar domains, is mainly involved in downstream signal transduction (Bentham et al., 2017).

The domain composition and architecture of pathogen receptor genes (PRGs) are important for the protein function. A domain is an evolutionary conserved entity because it has a specific functionality due to its fold. Thus, each conserved segment has a key role in protein function and folding (Moore et al., 2008). The two groups of PRGs share important characteristics and their activation promotes several common signaling pathways (Pruitt et al., 2021; Ngou et al., 2022). Both can trigger an immune response, including the activation of mitogen-activated protein kinase cascades, the production of reactive oxygen species, the increase in cytosolic calcium concentration and the expression of defense genes (Asai et al., 2008). In addition, NLRs can prompt a response that often culminates in a hypersensitive response (HR) (Dangl and Jones, 2001). It is worth noting that the RLP/RLK gene families result involved in several cellular processes, including growth, development and plant innate immunity (Andolfo et al., 2013), whilst NLRs are predominantly devoted to the activation of defense responses. The latter class showed a remarkable diversification within species and across species to meet specific needs (Barragan and Weigel, 2021). In addition, NLR gene signaling can rapidly augment the transcript and/or protein levels of key components of downstream immune response increasing the plasticity of innate immunity (Maruta et al., 2022).

Plant innate immunity originated for combating diverse and ever-evolving pathogens and the complex organization of its main players has an important role in its functioning. Here, we summarize the current view of the dynamics of NLR domain arrangement and the genomic architecture of plant defense arsenal on different scales, ranging from physical organization to transcriptional regulation. We describe links between genome organization and various genomic processes, such as the interplay between different PRGs. Finally, we provide an overview of the multilevel organization of innate immune response.

### Domain adaptation for promoting specific functions

The typical domains of NLR were already present in proteins of bacteria, protists, glaucophytes and red algae. In such organisms the NB is preferentially associated with domains like WD or beta-transducin repeats (WD40) or Tetratricopeptide repeat (TPR) domains to perform recognition/transduction activities (Andolfo et al., 2019). Several NB proteins with innovative domain combinations evolved in early plants. Independent NLR associations may have originated in Chlorophyta and in Charophyta algae (Sarris et al., 2016; Gao et al., 2018) by convergent evolution. An intriguing cross-species domain assembly between the NB domain and the LRR domain was highlighted in Charophyta unicellular green algae by Andolfo et al. (2019). The LRR-region of such genes showed high homology to RLPs, underpinning a putative cell-surface localization and an interconnected evolution history. Novel domain combinations have appeared, and the recombination of existing units has provided new functionalities. Best suited proteins with different cell locations from the plasma membrane (RLPs/RLKs) to cytoplasm (NLRs) have been employed for assembling a plant immunity network with the emergence of multicellularity. A burst of NB-LRR genes was observed in nonvascular plants (mosses, liverworts, and hornworts) mediated by reshuffling at the N- and C-terminal regions (Bornberg-Bauer et al., 2010; Sarris et al., 2016; Ortiz and Dodds, 2018). In vascular plants a large number of LRR encoding proteins, able to detect a variety of pathogens, was widespread in different species (Baggs et al., 2017). The structure and composition of such complex receptors have changed over time and the domain reorganization had an important role in evolutionary innovation (Urbach and Ausubel, 2017; Gao et al., 2018).

The ancient domain remodeling was further complicated by functional links connecting domains, supradomains and multidomains during the evolution of domain organization (Nepomnyachiy et al., 2017; Aziz and Caetano-Anollés, 2021). Basic principles of PRG domain composition emerged by comparing the distributions of the theoretical and observed domain association in 33 eudicots, highlighting that the 30% of possible combinations were missed, more than 60% of protein showed two or three domains but up to 20% were single domains (Sanseverino and Ercolano, 2012). Favorable protein conformations can be promoted by specific domain combinations. In addition, proteins including domains with a two-component response, such as DNA-binding activity linked to transcriptional regulation of responses to stressors and signal transduction systems, may have some benefits (Aziz and Caetano-Anollés, 2021).

The complex long-term coevolutionary arms race between plant and pathogens promoted species specific NLR combinations. For instance, TIR-NB-LRR (TNL) proteins are predominant in basal lineages and represent an important portion in the eudicot genomes but are absent in the monocots (Shao et al., 2016; Andolfo et al., 2017). A large reservoir of single domains or truncated NLR proteins is also scattered within resistance loci (Nishimura et al., 2017; Zhang et al., 2017).

The evolutionary trajectories of plant receptor genes have been extended through the addition of endogenous and exogenous functional domains, such as the C-terminal jelly- roll/Ig-like domains (C-JIDs), found in many TNLs, that directly interact with effectors (Ma et al., 2020; Martin et al., 2020), or integrated decoy (ID) domains that can bind pathogen effectors (Cesari et al., 2014; Kroj et al., 2016). Unraveling protein architecture and discovering local sequence conservation and diversification provides the key to understanding how proteins evolve (Konagurthu et al., 2021). For instance, evolution studies on plant NB domain showed that motif patterns are rearranged to acquire more tuned functions and to refine folding ability (Andolfo et al., 2020). Over an evolutionary timescale, the immune receptors are under a strong selection pressure for fixing functional advantages.

### Spatial genome organization of NB-LRR genes

Genomic-centric processes shaped the PRGs organization. NLR number can vary by orders of magnitude across different species with most plant genomes containing several hundred family members (Shao et al., 2016). Even closely related species can show lineage-specific mechanisms driving NLR expansions and contractions that reflect the plant lifestyle and the selection pressures derived from the environment (Tamborski and Krasileva, 2020), indicating that NLR evolution exhibits dynamic patterns of birth and death (Michelmore and Meyers, 1998). The NBS-LRR genes are not randomly distributed within plant genomes but rather are mainly organized in multi-gene clusters in hot-spot regions of plant genomes (Meyers et al., 1999; Hulbert et al., 2001; Richly et al., 2002; Zhou et al., 2004; Ameline-Torregrosa et al., 2008). NLR clusters can be divided into: homogenous clusters, including the same type of NLR, and heterogenous clusters containing diverse NLR classes. In addition, clusters containing a mixture of NLR, RLP and RLK were also found (Andolfo et al., 2013). Evolutionary forces governing gene clustering are not completely understood. The occurrence of gene duplication had great impact on expansion of this gene family in plant genomes *(* Baggs et al., 2017). Copy number variation likely maintains a diverse array of genes to retain advantageous resistance specificities (Jiang and Assis, 2017). Non-functional copies can evolve into functional alleles conferring disease resistance and changes in a pseudogene can lead to the gain of function. Individual NLR genes have also been associated with extreme allelic diversity as a consequence of point mutations enriched in surface-exposed regions of LRRs for acquiring new pathogen recognition capabilities by positive selection, inter-allelic and paralog recombination and domain fusions (Joshi and Nayak, 2013). On the other hand, large genomic deletion/insertion can provide the loss/gain of a specific gene family. Plant species arsenals are set up by the interplay of large-scale gene organization, that determines global conservation in the order of loci, and extensive local genome rearrangements mediated by recombination, tandem duplication, segmental and ectopic duplication, unequal crossovers, transposons, horizontal transfer and other reshuffling elements (Andolfo et al., 2021).

Adaptive diversification is induced by species-specific pathogen pressure thanks to the genome plasticity of plants (Mace et al., 2014; Di Donato et al., 2017; Mizuno et al., 2020). Regardless of the type of molecular mechanism, variations impact functionality and gene expression (Halter and Navarro, 2015). It seems that there is higher degree of association between genes in a cluster than just preferential co-localization. Recent studies showed that chromosomal regions with a defined gene density and activity, and with corresponding chromatin accessibility, histone modifications, and replication timing, are essential to orchestrate complex regulatory networks (Fritz et al., 2019). Each level of gene-genome intrinsic architecture is governed by mechanisms that we are just beginning to investigate (Nieri et al., 2017; Choi et al., 2018). An even more overwhelming challenge will be deciphering how PRGs are arranged, expressed and regulated within the three-dimensional (3-D) cellular context.

### Regulation of NB-LRR expression

The plant immune response must be highly plastic and strictly regulated, given the different types of pathogens to counteract and its fitness cost. In fact, plants not challenged by pathogen show a basal level of PRG expression that is able to monitor non-self-mediated changes in the plant cell while minimizes costs of expression (Richard et al., 2018; Borrelli et al., 2018). On the other hand, when attacked by pathogens a tight regulation of the immune transcriptome is essential for the activation of effective defense response (Karasov et al., 2017). The plant immune transcriptome is regulated by many different, interconnected mechanisms that can determine the rate at which genes are transcribed. Epigenetic modifications, such as DNA methylation (associated with actively transcribed genes), are required in the regulation of PRGs. Trimethylation of lysine 4 of histone H3 (H3K4me3), di- or trimethylation of H3K36 have been identified being epigenetic modifications essential for the defense response (Richard et al., 2018). In addition, ubiquitination of histones regulates the expression of NLR genes (Zou et al., 2014; Lai and Eulgem, 2018). Interestingly, areas of the genome featuring NLRs also frequently contain high densities of transposons (Miyao et al., 2003; Wei et al., 2016; Lai and Eulgem, 2018), which may attract epigenetic modifications to reduce transcription in the area. TFs, such as WRKY, are involved in PRGs regulation through the binding to W-boxes found generally enriched in promoter regions of NLR genes (Mohr et al., 2010; Richard et al., 2018). Small RNAs (sRNAs), including microRNAs (miRNAs) and small interference RNAs (siRNAs) (Waheed et al., 2021) function as negative regulators of NLR transcripts (Zhai et al., 2011; Li et al., 2012; Shivaprasad et al., 2012; Halter and Navarro, 2015).

The RNA surveillance pathways also have a leading role in the control of NLR-mediated resistance signaling. For example, nonsense-mediated mRNA decay (NMD) of NLR transcripts appears to play a role in defense induction similar to the miRNA/phasiRNA cascades. The perception of MAMPs can trigger transient suppression of NMD of NLR transcripts and, consequently, a temporary increase in NMD-targeted NLR transcripts, associated with enhanced disease resistance (Gloggnitzer et al., 2014). In addition, the nuclear RNA exosome regulates innate immunity in plants. For instance, mutations in components of the RNA exosome, which degrades RNAs in a 3′ to 5′ direction, suppress RPS6-dependent autoimmune phenotypes (Takagi et al., 2020). Alternative splicing can destabilize NLR transcripts triggering their own degradation and preventing their over accumulation (van Wersch et al., 2020). However, at least in some cases, alternative splicing can secure the synthesis of diverse transcript isoforms for full immunity (Jung et al., 2020). Recently published studies indicated that alternative polyadenylation (APA) of pre-mRNA is also an important regulatory mechanism of plant immune responses (Jia et al., 2017). APA can produce distinct transcript forms that differ in their coding sequences and in their 3’-untranslated regions, which are important for their function, stability, localization and translation efficiency of target RNA.

### Immune response networking

A complex network of interactions, based on intra- and inter-gene relationships, multilevel genome organization and DNA transcription and translation processes, regulates pathogen recognition events and defense responses. The defense mechanisms can be modulated through mutual interaction of a core set of receptors capable to activate the innate immunity responses (Saloman et al., 2020). The first discovered NLR-NLR cooperation dates to about twenty years ago, when it was discovered that two TNL genes, *RPP2A* and *RPP2B*, were required for resistance to downy mildew (Sinapidou et al., 2004). There are now many examples of NLR pairs, such as the Arabidopsis *RPS4*/*RRS1* and the rice *RGA4*/*RGA5* pairs (Cesari et al., 2014). One member (sensor) mimics the target of a pathogen effector, while the other member of the pair functions as a signaling ‘executor’ module that transduces the effector recognition. Moreover, it is emerging that many NLR-mediated immune responses require the presence and activity of so-called ‘helper’ NLRs, downstream signaling centers for a diverse array of sensor NLRs (Jubic et al., 2019). In this coupled reaction, sensor NLRs perceive effectors, and helper NLRs are involved in converting effector perception into immune activation (Cesari, 2018). Helpers are the Activated Disease Resistance 1 (*ADR1*), N Requirement Gene (*NRG1*) and NLR-REQUIRED FOR CELL DEATH (*NRC1*) (Gabriëls et al., 2007; Wu et al., 2016; Dong et al., 2016). Intriguingly, NRCs were first reported as required for the full function of transmembrane and cytoplasmic resistance receptors (Collier et al., 2011; Leibman-Markus et al., 2018). Functionally redundant *NRC* paralogs can display distinct specificities toward different sensor NLRs that confer immunity to oomycetes, bacteria, viruses, nematodes, and insects (Wu et al., 2017). The biochemical determinants that trigger helper-activation and physical interactions between sensor and helper remain unknown. Helpers could therefore act as ‘hubs’ to control signaling, guarding the whole immune signaling pathway rather than a specific molecule affected by an effector (Zhang et al., 2017; Leibman-Markus et al., 2018). Most likely, NLR helpers represent signal transduction and/or amplification levels that empower the innate immunity network (Wu et al., 2016). In addition, the plant pathogen immune response is promoted by the cooperation between the intracellular and extracellular receptors, even beyond early perception events (Yuan et al., 2021). A critical signaling component linking cell surface receptors and NLR-mediated immunity pathways is provided by reactive oxygen species produced by NADPH oxidase RBOHD (Yuan et al., 2021).

High-throughput gene expression data can provide reliable information for the inference of PRGs (Calle García et al., 2022). In two tomato-pathogen-specific interactions, different networks of PRGs acting in concert were found (Andolfo et al., 2014). Although, plant immunity shares the same signaling mechanisms, the rewiring of PRGs networks may promote connection changes among defense pathways in specific plant-pathogen interactions. Investigation of differentially regulated PRGs could lead to the identification of pathogen-specific response patterns. Multiple responses can be merged into a single network model for capturing all the possible dynamic trajectories.

### Organization of immune responses

**Figure 1:**
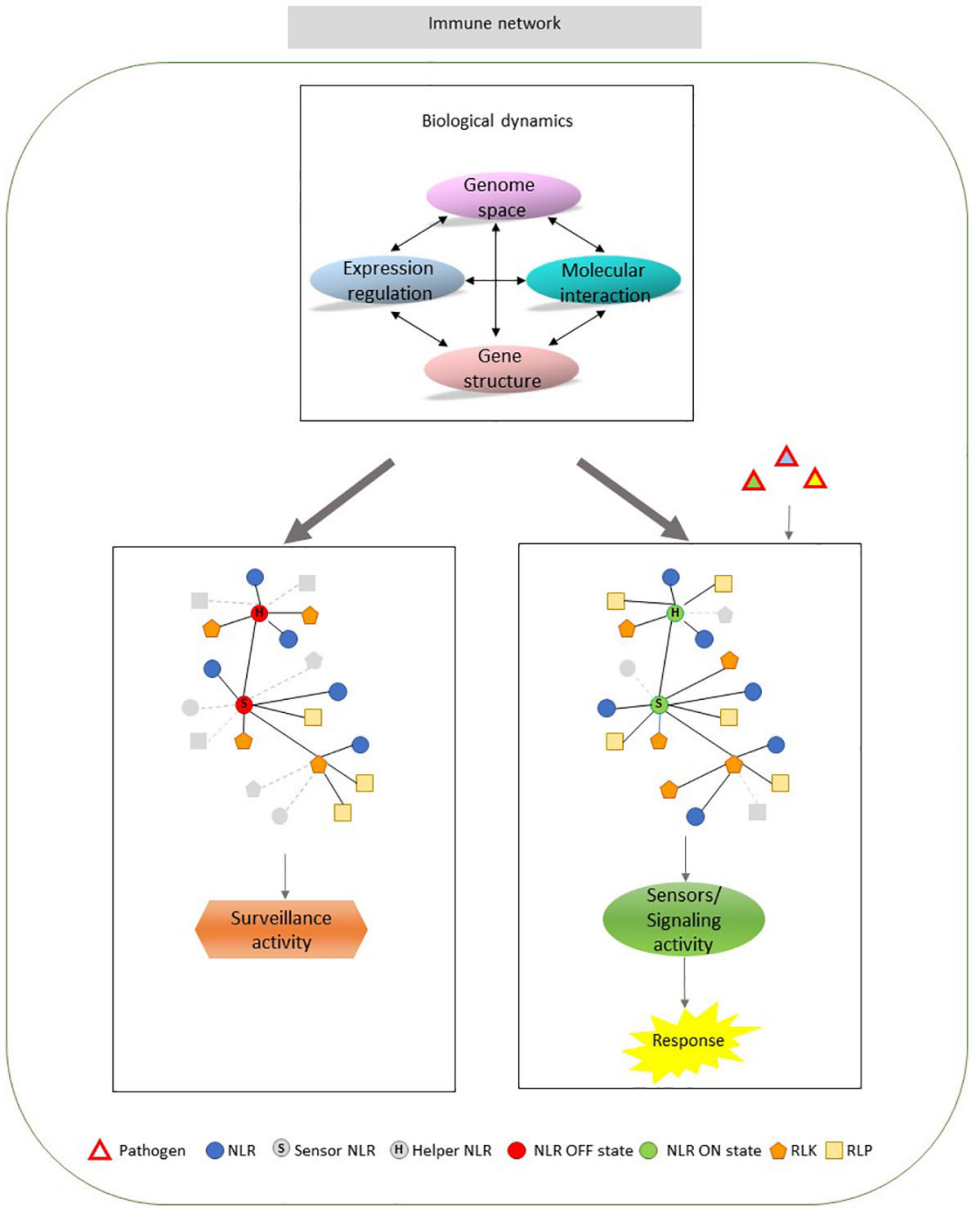
Organizational overview of plant innate immune system. Upper middle panel. The immune system functioning is modulated by a fine control over the pathogen receptor genes (PRGs) activities at different levels (ovals). They include gene structure, genome space, molecular interaction and gene expression regulation. The connections among the different levels show a very complex and dynamic arrangement (indicated by arrows). All the potential levels can determine the loss/gain of connections in PRG network. Left panel. PRGs network organization in plants not challenged by pathogen. NLRs are in a resting state (OFF state) resulting in a PRG surveillance activity to recognize potential pathogen infection. Right panel. PRGs network organization challenged by pathogen. The activation of a sensor NLR and a potential helper (ON state) can initiate a response culminating in innate immunity. The network edges represent every kind of biological interaction influencing the member activity. Gray color indicated the changing connections among members of each network. NLR, nucleotide-binding leucine-rich repeat proteins; RLK, receptor-like kinase; RLP, receptor-like protein.

Defense scenarios can be depicted taking into account: the layer of defense, direct and indirect interaction, the network of response, cell sensing of pathogen and fitness needs (Andolfo and Ercolano, 2015). NLRs are involved in both perception and activation of immune signaling. Recent breakthroughs are starting to disclose mechanisms by which NLRs initiate immune signaling after effector perception. Conformational changes lead to the exchange of ADP by ATP and the oligomerization induction with the establishment of a functional ‘resistosome’ (Burdett et al., 2019). Complex formation, self-association or heteroligomerization was shown to be important for the activity of many NLRs (Casey et al., 2016; Zhang et al., 2017; Li et al., 2020; Jacob et al., 2022). Understanding how molecular entities evolve, work and are interconnected in any biological process is crucial. The high plasticity governing the innate immune system is founded on a complex functional architecture and a multi-level control as proposed in the **Figure 1** model. Multiple levels, including gene structure, genome-gene relationships, gene regulation, molecular interaction show highly dynamic connections (**Figure 1** upper middle panel) that are able to regulate the innate immunity receptors with a different output, surveillance or defense response (**Figure 1** left and right panels). In particular, within this model, PRGs are involved in a “multi-actors” system, including NLRs that may act as sensors or “helpers” (**Figure 1** left and right panels). Leading sensors are able to coordinate a response, which may include the activity of different PRG groups (Andolfo et al., 2014; Ngou et al., 2021). The understanding of network structure, considering the distribution of the interaction strength, the challenges for the establishment of these interactions and the corresponding effects could be highlighted by a decomposition approach. It would be interesting to dissect the whole set of molecular interactions across the different levels, to identify the role and the spatial distribution of each element. Current knowledge of the immune network system is still limited and can be improved by studying its structural properties. Pathogen receptor system is continuously shaped over time to find its optimal arrangement thanks to different biological dynamics.

## Author contributions

MRE, conceived the study and was primarily involved in writing the manuscript and in producing the figure. DD, substantially contributed to the writing and revising of the manuscript and was centrally involved in figure design. GA, contributed to the writing of immune response networking paragraph and revised the manuscript. LF, revised the manuscript and coordinated the work. All authors contributed to the article and approved the submitted version.

## Funding

This work was supported by the Ministry of University and Research and carried out within the Harnesstom Project (101000716) funded by the European Community.

## Conflict of interest

The authors declare that the research was conducted in the absence of any commercial or financial relationships that could be construed as a potential conflict of interest.

## Publisher’s note

All claims expressed in this article are solely those of the authors and do not necessarily represent those of their affiliated organizations, or those of the publisher, the editors and the reviewers. Any product that may be evaluated in this article, or claim that may be made by its manufacturer, is not guaranteed or endorsed by the publisher.
